# Mobbing as a source of psychological harm in workers

**DOI:** 10.47626/1679-4435-2022-766

**Published:** 2023-02-13

**Authors:** Jenifer Grotto-de-Souza, Hildegard Hedwig Pohl, Deryck Aguiar-Ribeiro

**Affiliations:** 1 Departamento de Ciências da Vida, Universidade de Santa Cruz do Sul, Santa Cruz do Sul, RS, Brazil

**Keywords:** workplace, workplace violence, bullying, ambiente de trabalho, violência no trabalho, bullying

## Abstract

Workplace harassment is a phenomenon as old as work itself. It constitutes a form of discrimination that violates labor laws and civil rights, a type of silent violence that affects work relationships, destabilizes the victim, and harms the physical and mental health of workers. The present study aimed to investigate the association between psychological harm and workplace mobbing through a descriptive narrative review of the literature. PubMed and Scopus databases were searched in July and August 2020 using the following Health Sciences Descriptors: “Harassment, Non-Sexual”, “Workplace Violence”, and “Working Environment”. Inclusion criteria were full-text articles written in English and published between 2015-2020. Thirty-three articles were preselected, of which 17 were excluded because they did not meet the inclusion criteria. Sixteen articles were included in the study. Globalization, in association with increased competitiveness in the work environment, has promoted a continuous and progressive deterioration of work relationships, which has been aggravated by the expansion of communication technologies and social media. The frequency of workplace mobbing and its consequences on the income and quality of life of workers has increased. The magnitude of the association between harassment and psychological harm is still underestimated due to low reporting rates, which are motivated by the trivialization of toxic work relationships. Regardless of how mobbing occurs in the workplace, it negatively affects the physical and mental health of workers, sometimes even leading to permanent disability.

## Abstract (pt)

O assédio no trabalho é um fenômeno tão antigo quanto o próprio trabalho. Consiste em discriminação que viola leis trabalhistas e direitos civis, uma violência silenciosa que afeta as relações de trabalho e desestabiliza a vítima, abalando a saúde física e mental. O presente estudo teve por objetivo correlacionar o adoecimento psíquico do trabalhador com o assédio moral no trabalho através de uma revisão narrativa da literatura, de caráter descritivo. Realizou-se uma pesquisa nas bases de dados PubMed e Scopus em Julho e Agosto de 2020, mediada pelos seguintes Descritores em Ciências da Saúde: “*harassment, non-sexual*”, “*workplace violence*” e “*working environment*”. Foram selecionados artigos em língua inglesa, escritos entre 2015-2020, em sua versão final completa no momento da pesquisa. Foram pré-selecionados 33 artigos, dos quais 17 foram excluídos por não se enquadrarem nos critérios anteriores, permanecendo 16 artigos para apreciação final. Observou-se que a globalização, em conjunto com a crescente competitividade no ambiente de trabalho, levou a uma deterioração contínua e progressiva das relações de trabalho, agravada pela expansão das tecnologias de comunicação e mídias sociais. Percebe-se um aumento na frequência do assédio moral e nas suas consequências no rendimento e na qualidade de vida do trabalhador. A magnitude da associação entre assédio e adoecimento do trabalhador ainda é subestimada devido ao baixo índice de denúncias, motivado pela banalização de relações tóxicas de trabalho. Fica claro que, independentemente do modo como o assédio moral ocorre no ambiente de trabalho, ele afeta negativamente a saúde física e mental do trabalhador, podendo levar a uma incapacidade permanente.

## INTRODUCTION

Although workplace harassment is as old as work itself, it became the subject of scientific studies and reported more frequently only from the 1980s onwards.^1^ According to the International Labor Organization (ILO), workplace harassment refers to unacceptable practices and behaviors, threats, or intimidations that occur in one or more occasions and can result in physical, psychological, sexual, or economic harm. It may also constitute a form of gender-based violence.^2^

Workplace harassment is a form of discrimination that violates a wide range of labor laws and civil rights. It is an offensive conduct that can be considered intimidating, hostile, or abusive and that the worker must often deal with to keep their job. Although allegations have increased in recent years, reports are still infrequent, largely due to the trivialization of harassment, in which these situations are seen as routine and not understood as harmful. Recently, with the expansion of digital media, harassment has been frequently reported through electronic communications. This wide range of presentations requires companies and organizations to be constantly vigilant to avoid all forms of harassment.^3^

Mobbing is a silent form of violence that affects work relationships, destabilizes the victim, and harms the physical and mental health of workers. It can cause moral, psychological, physical, and material harm, as well as temporary or permanent disability. Thus, mobbing is a topic of legal, social, and psychological importance in the work environment.^1^ Although there are few formal complaints and related lawsuits, studies evaluating the frequency of harassment in different work environments have shown that most workers have already experienced mobbing, which negatively impacted their professional and personal lives.^4-6^

Recognizing the importance of the topic, the ILO published the Convention Concerning the Elimination of Violence and Harassment in the World of Work. The document recognizes everyone’s right to work in an environment free of violence and harassment and the importance of a work culture based on mutual respect and human dignity. It also states that violence and harassment in the workplace can constitute a human rights violation or abuse that is a threat to equal opportunities, unacceptable, and incompatible with decent work.^2^

Considering the aforementioned, the present study aimed to investigate the association between psychological harm in workers and workplace mobbing.

## METHODS

This study consisted of a descriptive, narrative review of the literature. PubMed and Scopus databases were searched in July and August 2020 using the following Health Sciences Descriptors: harassment, non-sexual; workplace violence; working environment. Inclusion criteria were full-text original articles published in English between 2015-2020 about workplace mobbing and its relationship with the mental health of workers. Initially, 33 articles were preselected, of which 17 were excluded for not meeting the inclusion criteria. Sixteen articles were included after the eligibility criteria were applied.

### WORK: JUST A MEANS OF SUBSISTENCE?

Work plays a central role in shaping human identity. Therefore, understanding its significance and the degree of affection between workers and their jobs is of great importance for understanding the relationship between man and work, considering that people spent most of their time working.^7^ In the ontological sense, work is related to the interaction that people establish with nature; an activity with a social objective that is, at the same time, conscious. Work is transformative because it allows man to transform natural products into means of subsistence, but is also productive because it generates goods, services, and relationships in its construction process.^8^ Work changes interpersonal relationships by promoting bonds, interactions, and exchanges of experience, in addition to having a positive psychosocial impact by affirming one’s culture and revealing one’s identity and achievements. Therefore, work cannot be only considered a means of subsistence.^9^

In many countries, workplace violence is a common problem that often lacks adequate reporting. However, estimates from the Crime Survey for England and Wales suggest that approximately 1.4% of adult workers suffered some form of workplace violence between 2011 and 2012, of whom 0.7% suffered physical violence and 0.8% were verbally attacked. In gross numbers, this percentage accounts for 312,000 victims, consisting of 159,000 aggressions and 169,000 threats in this short period of time.^6^

For many years, work was not seen as part of the relevant aspects of human life, of the way of being, or as a source of psychological suffering. Organic, genetic, or family aspects were seen as plausible justifications for the mental health disorders developed by workers. Despite the increase in the number of studies on this subject, it is part of our culture to maintain a distance between the themes of worker’s health and mental health, as if both could not be interrelated.^10,11^

Labor relations have undergone major changes over time. In capitalist society, the worker undergoes a process of alienation, in which work and its product are no longer controlled by the worker, but rather control the worker instead. Today, work tends to add less meaningful elements to people’s lives, and may be modified into something forced or imposed, that is, a source of suffering. Flexible accumulation, which is a part of capitalism, takes work beyond the working environment, involving everyday dimensions of life such as leisure and rest time and turning one’s entire existence into an extension of work.^12^ In addition, work relationships are increasingly deteriorating, labor laws are becoming more flexible, and the rates of outsourcing, unemployment, and underemployment are rising. This creates a fruitful environment for psychological and physical harm.^13^

Social medicine in Latin America, with the goal of understanding the complexity of the human psychic nexus, developed categories of workload based on elements of the work process that are interrelated and related to the body and psyche of the worker and that can generate loss of potential capacity and wear. Physical, chemical, biological, mechanical, physiological, and psychological loads act on the worker, complementing each other and resulting in physiological changes.^14,15^

### WORKPLACE MOBBING: A NEW EPIDEMIC?

Workplace mobbing is almost as old as work itself. However, harassment rates have been increasing and gaining more visibility due to changes in work environments and the expansion of computerization and communication. Workplace violence is defined as any event in which someone is abused, threatened, or attacked in work-related circumstances and is seen as a public health problem. It may be physical, moral, or psychological in nature and may be carried out by employers, coworkers, clients, or partners. Although highly underreported, violence is a common phenomenon.^6^ Mobbing is one of the types of workplace violence. It is defined as any abusive, repetitive, and systematic conduct that compromises the dignity and physical or mental integrity of the person. The conceptualization of mobbing was developed in the context of globalization, which increasingly exposes workers to excessive demands, organizational restrictions, and work intensification.^16^

Mobbing is prevalent in the most diverse settings, and no professional category seems to be spared. In the prison system, a study estimated that 28% of jailers had suffered some type of harassment, with their coworkers being responsible for the violent act.^6^ Health care services are fertile environments for exposure not only to biological and ergonomic risks, but also to mobbing, with nursing professionals often identified as a vulnerable group to this type of violence.^4^ Medical school is also frequently cited as a source of harassment. The constantly hostile environment can lead to mental exhaustion and harm, with increasingly high rates of depression and suicide among medical residents.^17^ In factories, hostile behavior is frequently observed, and harassment tends to be carried out by hierarchical superiors or coworkers.^16^

Due to policies to reduce costs related to human resources, companies started to hire “multitasking professionals”, who perform functions formerly occupied by two or three workers. The worker is often displaced from the function for which he or she was hired, generating a breach of expectations and excessive demands. At the same time, the worker is forced to carry out these activities to keep their job. This type of work environment promotes mobbing and other forms of violence, especially when leaders are not qualified to do their job.^18^

With the expansion of technology and computerization, harassment has crossed the boundaries of the work environment and has invaded the private space. Messaging applications provide a platform for harassment, allowing excessive demands when workers are no longer on their paid duty hours and occupying part of their leisure and rest time. At the same time, the use of these applications allows the documentation of abusive behavior, providing a basis for legal accountability of the aggressor.^19^

The policy adopted by companies plays an important role in the development and containment of mobbing. Higher-paid workers are less exposed to harassment and a culture of silence. By adopting policies that facilitate reporting, companies tend to, in addition to breaking the silence of victims, provide fewer opportunities for harassers.^20^ To stop workplace mobbing, it is often necessary to change the company’s culture. In medical education, for example, learning through humiliation used to be considered a normal educational practice, as if it constituted a rite of passage. Today, it has been recognized as harassment, and many hospitals have proposed more standardized teaching methods.^21^

### MOBBING AS A SOURCE OF PSYCHOLOGICAL HARM

Work, in addition to shaping identity, can be a source of psychological suffering and discomfort, especially when the worker does not feel valued, faces a hostile or unsafe environment, or when the job does not provide perspectives or is not consistent with the worker’s personal expectations.^18^ The work environment consists of systems, situations, and conditions in which workers performs their tasks. Toxic environments provide repulsive experiences that lead to poor work performance, acting as cancers that harm the entire organism. Toxic behaviors may increase organizational costs because, in addition to harming the company’s image, they cause low self-esteem among workers, high turnover, absenteeism, and reduced productivity.^22,23^ Business management should include the discussion of ethics and morals and should recognize the importance of the work environment for the proper development of work activities.^24^

Victims of mobbing tend to become isolated in the workplace, as coworkers tend to stay quiet regarding the situation either for fear of becoming a victim of the aggressor or due to the relationship the aggressor has established with the rest of the team. People tend to protect those with whom they have personal relationships when they commit moral infractions like theft and harassment. This trend is independent of gender, moral foundations, or sensitivity. This situation intensifies the negative impact on the victim, since isolation and the feeling of loneliness often prevents the victim from reporting the aggression, thus perpetuating the cycle of harassment.^25,26^

The positive meaning workers attribute to their activities may become negative when the company’s requirements and beliefs are inconsistent with their personal values. The perception of lack of appreciation of their productivity and dedication, associated with the lack of financial incentive, puts the worker in a vulnerable situation, breaking the psychological contract. In the long term, this structure results in recurrent absences, turnover, worsening performance, dissatisfaction, and a lower sense of well-being, predisposing workers to depressive episodes.^18^

Harassment can cause or aggravate many psychological and behavioral disorders. Most violent situations are experienced silently, which significantly interferes with the real knowledge of the correlation between harassment and psychological harm. Either because of the fear of losing one’s job or because of the trivialization of harassment in the structure of some organizations, harassment is invisible, although everyone can see the damage it causes. The degree of psychological harm depends on the support received by the worker, as well as on their personal capacity for endurance.^7^

## CONCLUSIONS

With the general globalization of means, services, and access to information and technology, associated with the growing and fierce competition in the work environment, there has been a continuous and progressive deterioration of labor relations in recent years, aggravated by the expansion of communication technologies and social media. In addition, there has been an increase in the frequency of mobbing, whose social costs are immeasurable, since it impacts productivity and frequency at work, in addition to causing significant harm to the mental health of the worker. The magnitude of the association between harassment and psychological harm is still underestimated due to low reporting rates, motivated by the trivialization of toxic work relationships. To change this reality, the structure of organizations and the work culture should undergo drastic transformations, a long process that requires collaboration from everyone involved.
